# A Comprehensive Review: Advances in Mesenchymal Stem Cell Applications for Burn Wound Repair

**DOI:** 10.1155/sci/6683745

**Published:** 2025-03-20

**Authors:** Hui-Juan Zhang, Jing-Jie Ming, Hong-Xiao Zhang, Shao-YI-Han Fang, Quan-Wen Liu, Hong-Yan Zhang

**Affiliations:** ^1^Medical Center of Burn Plastic and Wound Repair, The First Affiliated Hospital, Jiangxi Medical College, Nanchang University, Nanchang 330031, China; ^2^The National Engineering Research Center for Bioengineering Drugs and the Technologies, Institute of Translational Medicine, Nanchang University, Nanchang 330031, China; ^3^Jiangxi Provincial Key Laboratory of Interdisciplinary Science, Nanchang University, Nanchang 330031, China

**Keywords:** mesenchymal stem cells, mechanism, paracrine effects, transdifferentiation, wound healing

## Abstract

Tissue repair following skin injury is a complex process that encompasses hemostasis, inflammation, tissue cell proliferation, and structural remodeling. Mesenchymal stem cells (MSCs) are derived from the mesodermal layer of tissues and possess multidirectional differentiation potential and self-renewal capabilities. MSCs from various sources, including the bone marrow, adipose tissue, dental pulp, umbilical cord, and amniotic membrane, have demonstrated effectiveness in promoting skin injury repair. They aid in this process by fostering the formation of new blood vessels in damaged tissues, self-renewal, or transdifferentiation into skin or sweat gland cells. Moreover, MSCs promote the proliferation and migration of skin cells, reduce wound inflammation, and restore the extracellular matrix through paracrine secretion. In this paper, we review recent findings regarding MSCs and their role in burn wound repair. Additionally, we explore the potential of combining MSCs with various biomaterials for treating burn wounds and analyze clinical cases wherein MSCs were administered to patients, offering insights into ongoing research on MSC-based therapies for skin injuries.

## 1. Introduction

Burn injuries have devastating consequences, impacting not only the physical well-being and emotional equilibrium of patients but also their overall quality of life [1]. Burns rank as the fourth most frequent injury worldwide, followed by traffic accidents, falls, and deliberate harm [2]. Annually, ~6 million hospitalizations result from burn injuries [3]. Despite the advancements in technology that have reduced burn-related fatalities, managing and treating burn wounds remains a challenging task [1–3].

Burn injuries lead to tissue damage resulting from external agents such as chemicals, heat, and electrical currents [4]. Based on severity, depth, extent, body location, and overall clinical outcome, burns are categorized using a four-degree scale. First-degree and superficial second-degree burns primarily involve the epidermis and part of the dermis. Superficial first- and second-degree burns primarily involve the epidermis and a portion of the dermis, healing rapidly within 1–2 weeks, usually without scarring. In contrast, deep second- and third-degree burns affect a major part or the entire dermis and deeper tissues, requiring an extended healing time and carrying elevated risk of infection, organ dysfunction, and scar formation [5]. Severe burns trigger systemic inflammatory response syndrome (SIRS), resulting in increased capillary permeability and vascular resistance, ultimately contributing to elevated mortality and morbidity rates in patients [6]. Despite various burn management approaches, ranging from surgical interventions to intensive care and wound treatment, substantial challenges persist.

Mesenchymal stem cells (MSCs), which exhibit fibroblast-like properties and adhere to plastic surfaces, possess a remarkable ability to self-renew and differentiate into various cell lineages. Bone marrow (BM)–MSCs, adipose-derived mesenchymal stem cells (ADSCs), and umbilical cord stem cells (UC-MSCs) originate primarily from the BM, adipose tissue, and UC blood, respectively. MSCs have revolutionized the treatment landscape for various human ailments, including diabetes, heart disease, multiple sclerosis, and graft-versus-host disease (GVHD) [7]. In burn treatment, MSCs play a crucial role in wound healing. In this paper, we provide an extensive overview of burn wound healing biology and pathology, along with preclinical and clinical trials exploring various types of MSCs. We also discuss and analyze the major challenges that must be addressed before MSCs can be widely adopted for the treatment of burn wounds. Furthermore, we propose a potential strategy for burn treatment using stem cells that can serve as a reference for future research and clinical applications.

## 2. Development of MSCs

Friedenstein, Chailakhyan, and Gerasimov [8] discovered a nonhematopoietic population of BM stromal cells, similar to fibroblasts, within the BM. In 1992, Arnold Caplan officially coined the term MSCs, considering their ability to differentiate into multilineages [9]. Subsequently, the International Society for Cellular Therapy (ISCT) established standardized criteria for defining MSCs. These criteria include (1) adherent growth; (2) expression of specific cell surface antigens (high expression of CD105, CD73, and CD90 but no expression of CD45, CD34, CD14, CD79, and HLA-DR); (3) ability to differentiate into adipocytes, osteoblasts, and chondrocytes [10]. Recently, MSCs have garnered attention because of their ample availability, accessibility, ethical neutrality, self-renewal capacity, and potential for multilineage differentiation [11]. MSCs substantially contribute to wound healing and represent an alternative treatment for burn injuries [12].

## 3. Physiological Characteristics and Treatment of Burns

The multifaceted phenomenon of burn wound healing involves intricate interactions among diverse cell types, including epidermal and dermal cells, neoangiogenesis, extracellular matrix (ECM), and plasma-derived proteins. These processes are regulated by cytokines and growth factors [13]. The healing trajectory unfolds through three primary interwoven phases—inflammation, proliferation, and remodeling [14]. During the initial burn stages, inflammation occurs as nearby capillaries and small blood vessels constrict, diminishing the blood flow. Platelet aggregation at the wound site results in clot formation, and the release of vasoactive substances and growth factors exacerbates vasoconstriction and further impedes blood flow. These events activate endogenous and exogenous coagulation mechanisms, ultimately reducing bleeding [15]. Fibroblasts play a crucial role in wound healing as the primary functional cells responsible for granulation tissue formation and wound contracture. These versatile cells can originate from the injury site or synthesize and secrete collagen as part of the inflammatory response, making them essential for effective wound repair [16]. Meanwhile, epidermal cells at the periphery of the wound and base migrate and proliferate, sealing the epithelial gap after injury and progressively enveloping the wound surface [17]. Granulation tissue exhibits sustained proliferation and transforms into scar tissue over an extended period. Fibroblasts play a pivotal role in wound healing. Growth factors such as platelet-derived growth factor (PDGF), epidermal growth factor (EGF), fibroblast growth factor (FGF), insulin-like growth factor (IGF), and fibroblasts transition into myofibroblasts and synergize with macrophages, mast cells, and lymphocytes to enhance wound healing [18]. Fibroblasts contribute to the wound healing process by participating in granulation tissue formation and synthesizing and secreting collagen and ECM components. Excessive or prolonged edema and inflammation exacerbate pain and impair wound healing [19]. Conventional burn wound management approaches include debridement, anti-infection protocols, negative-pressure drainage, biological dressings, skin grafts, flap grafts, and other strategies [20]. Although mortality rates in patients with burns have reduced due to ongoing advancements, challenges such as burn wound chronicity, scar formation, and impaired sweating in healed skin are still present. Therefore, scientists and clinicians are constantly trying to minimize scarring and restore normal skin structure and function [21].

## 4. Role of MSCs in Burn Wound Healing

MSCs expedite burn wound recovery by enhancing wound closure, stimulating angiogenesis, suppressing inflammation, modulating ECM remodeling, preventing apoptosis, and promoting cell proliferation.

### 4.1. MSCs Promote Tissue Repair Through Self-Renewal and Differentiation

The skin consists of epidermal, dermal, and subcutaneous layers. First- and second-degree burns affect the epidermis down to the deep dermal layer, whereas third-degree burns extend throughout the skin, including the subcutaneous tissue. While conventional techniques are sufficient for minor skin injuries, innovative strategies are required to enhance wound healing in severe burns. MSCs are a class of adult stem cells with the potential for self-replication and multidirectional differentiation, not only into related cells of homologous origin but also into other germ layers. In laboratory settings (in vitro), the addition of growth factors or specific culture media, such as indomethacin, hydrocortisone, and transforming growth factor-*β* (TGF-*β*), guides MSCs toward proliferation and differentiation along various phenotypic pathways. Conversely, in living organisms (in vivo), tissue damage-related signals, such as trauma, fractures, inflammation, necrosis, and tumors, directly mobilize MSCs, prompting their differentiation into connective tissue cells [22]. Recombination signal binding protein for immunoglobulin kappa J region (RBP-JK) is a transcription factor that impedes the differentiation of MSCs. Luo et al. [23] silenced the expression of RBP-JK and facilitated BM-MSCs conversion into vascular endothelial cells. Mammals must meticulously regulate their core body temperature owing to the susceptibility of tissues and organs, particularly the brain, due to overheating. Abundant endocrine glands enable humans to efficiently dissipate surplus heat. Individuals with impaired sweating function face the risk of heatstroke and even mortality. Despite the therapeutic potential of endogenous regeneration, the widespread use of sweat gland (SG) progenitor cells is limited by their inadequate regenerative capacity and susceptibility to disturbances in adjacent niches caused by wounds. Consequently, there is an urgent need to devise innovative and effective strategies for the regeneration of SGs. Studies have demonstrated that BM-MSCs can differentiate into sweat gland-like cells (SGCs) in vitro and display secretory functions upon transplantation into living organisms in vivo [24]. In their study, Sun et al. [25] effectively reprogrammed BM-MSCs into SGCs by specifically targeting the ectodysplasin (EDA) promoter using CRISPR/dCas9-effector (dCas9-E).

### 4.2. MSCs Promote Neovascularization to Facilitate Skin Damage Repair

Angiogenesis plays a pivotal role in wound healing, with neovascularization providing essential oxygen and nutrients for the maturation of granulation tissue at the wound site [26]. MSCs contain a diverse array of regulatory molecules, including vascular endothelial growth factor (VEGF), FGF, TGF, tumor necrosis factor (TNF), angiopoietin, and interleukin (IL-8). These factors collaboratively facilitate vascular remodeling in burn wounds and accelerate the intricate wound healing process [27]. In rat burn wounds, administration of ADSCs led to heightened VEGF expression and enhanced angiogenesis, positively impacting wound healing—a discovery made by Zhou et al. [28]. Additionally, Rezaei et al. [29] observed that ADSC transplantation in burned rat wounds upregulated TGF-*β* and VEGF, resulting in markedly increased neovascularization compared with that in the control group—an enhancement that significantly improved wound healing in severely burned rats. Zhu et al. [30] demonstrated that the intravenous injection of BM-MSCs into rat tail veins robustly activated the Notch signaling pathway, leading to pronounced neovascularization. Loder et al. [31] revealed that ASC (Adipose-derived stem cells) transplantation into third-degree burn wounds in mice promptly triggers an early biochemical surge in neovascularization. Furthermore, Nie et al. [32] emphasized that in damaged skin tissue, ADSCs significantly boost the secretion of essential growth factors, including FGF, VEGF, and HGF. This enhancement facilitates vascular regeneration and expedited wound healing. Diao et al. [33] revealed that VEGF, beyond its direct role in angiogenesis, activates transcription factors to orchestrate endothelial progenitor cell (EPC) regulation, EPC recruitment to the BM, and inhibition of EPC apoptosis, collectively promoting effective wound healing. These findings suggest that MSCs can enhance angiogenesis at the injury site, both directly and indirectly, by releasing paracrine growth factors. This ultimately improves blood circulation and facilitates wound healing.

### 4.3. MSCs Modulate the Immune Response and Inflammation to Promote Repair of Skin Damage

The inflammatory response of the body serves as a crucial self-defense mechanism. Following tissue damage, a controlled inflammatory response facilitates self-protection and repair. However, excessive inflammation can hinder wound healing. MSCs possess homing abilities that direct them to migrate to the sites of skin injury and inflammation [34]. During the inflammatory response, proinflammatory factors (including IL-1*β*, IFN*γ*, and TNF*α*) activate signaling pathways within the MSCs. This activation promotes the paracrine secretion of key anti-inflammatory proteins, such as TNF-*α* and other proinflammatory cytokines from resident macrophages stimulated gene/protein 6 (TSG-6) [35]. Consequently, the migratory chemotaxis of immune cells, specifically that of neutrophils and macrophages, is inhibited. Additionally, MSCs suppress dendritic cell maturation and activation while reducing the proliferation and activation of NK cells, B cells, and T cells. Furthermore, MSCs exert potent anti-inflammatory effects by upregulating proteins such as IL-4, IL-10, and IL-13 while downregulating proinflammatory factors, such as TNF-*α*, IL-1*β*, IL-6, IFN-*γ*, and iNOS [36–38]. Activation of MSCs is reportedly associated with increased regulatory T-cell (T-reg cell) generation and reduced cytotoxic effects of CTLs [38]. Consequently, this modulation contributes to a diminished inflammatory response in wounds. Additionally, MSCs interact with macrophages, shifting their internal programming from a proinflammatory M1 phenotype to an anti-inflammatory state. These interactions lead to elevated expression of surface markers associated with activated M2 macrophages, including CD206, phagocytosis genes, and key anti-inflammatory factors [39]. Regmi et al. [40] revealed that MSCs promoted burn wound healing by modulating the immune response and facilitating the release of inflammatory factors. Zhao et al. [41] proposed that the extracellular vesicles (EVs) derived from MSCs mitigate mitochondrial damage and inflammation by stabilizing mitochondrial DNA. Rangatchew et al. [42] found that MSCs significantly reduced inflammation and accelerated the healing rate of acute burns. In a rat model of deep dermal burns, allogeneic MSCs have demonstrated regenerative potential and enhanced immunomodulation. Transplantation of MSCs in burned rats significantly reduced the serum levels of leukocytes, C-reactive protein, TNF-*α*, IL-6, and IL-10. This reduction suggests that MSC transplantation inhibits the production of inflammatory factors during the postburn period, thereby mitigating the inflammatory function of MSCs. Yagi et al. [43] further supported this finding by showing that BM-MSCs alleviated systemic inflammatory responses and the organ damage caused by burn injuries in a 30% burn rat model. The anti-inflammatory properties of MSCs significantly improve the inflammatory microenvironment in burn wounds, thereby accelerating wound healing.

### 4.4. Role of MSC-Derived Exosomes in Skin Wound Repair

Despite the benefits of MSCs and their proven therapeutic efficacy in burn wounds, the biosafety risk associated with the in vivo infusion of living cells, such as microvascular obstruction and acute inflammatory reactions, needs to be examined. This prevailing view posits that the therapeutic effects of MSCs primarily depend on their paracrine factors, which are predominantly transported via EVs and subsequently released into the extracellular milieu. Based on their size, source, and release pathway, EVs fall into two primary categories—microvesicles and exosomes. Cup-shaped, lipid bilayer-enclosed biological nanoparticles, measuring 30–150 nm, and originate from luminal vesicles within intracellular bodies at late stages. These exosomes bud inward through the intracellular membrane and exit the cells upon the fusion of their outer membrane with the plasma membrane. MSC-EXOs contain abundant biologically active molecules including proteins and nucleic acids. These exosomes play a crucial role in modulating growth factor expression and activating signaling pathways [44]. Moreover, cell proliferation, migration, and angiogenesis are governed by regulators such as phosphatidylinositol 3-kinase/protein kinase B (PI3K/AKT), the extracellular signal-regulated kinase (ERK) cascade, and Wnt/*β*-catenin. Thus, these processes promote epithelial regeneration [45]. Qian et al. [46] demonstrated that ADSC-EXOs enhance skin wound healing in mice by activating the Wnt/*β*-catenin pathway through upregulation of SOX9. This activation leads to increased proliferation, migration, and invasion [46]. Exosomes mitigate unforeseen safety concerns related to immune rejection, genetic transmission, disease transmission, and ectopic cell differentiation. Moreover, issues present in whole-cell therapies, such as MSC-EXOs, have emerged as a promising therapeutic approach for various human diseases. Numerous preclinical studies have evaluated the therapeutic potential of MSC-EXOs for burn wound healing. For instance, the intravenous administration of exosomes released by human UC-MSCs in a rat model of third-degree burns significantly improved wound healing by reducing inflammatory factors [47]. Moreover, intravenous administration of exosomes derived from human UC-MSCs to a rat model with severe burn-induced acute lung injury (ALI), commonly referred to as hUC-MSC-EXOs, significantly reduced TNF-*α*, IL-1*β*, and IL-6 levels, resulting in effective mitigation of ALI. Thus, further investigation into its therapeutic potential for diverse human diseases is necessary [48]. Furthermore, local administration of exosome A, derived from human induced pluripotent stem cells (iPSCs), near the wound site in mice with deep second-degree burns significantly enhanced traumatic injury healing. This effect was attributed to the facilitated migration of endothelial cells and keratinocytes, which ultimately promoted burn wound recovery [49]. Notably, administering exosomes released by ADSCs directly into mouse skin wounds with extensive lesions effectively curtailed proliferative scar formation. This outcome was achieved by modulating collagen deposition and inhibiting fibroblast-to-myofibroblast transdifferentiation, ultimately reducing scar formation [50]. Harnessing the potential of MSC-EXOs shows great promise as a therapeutic strategy for enhancing burn wound healing.

## 5. Application Strategies for MSCs

In recent years, MSCs have emerged as an innovative therapeutic approach for wound healing and tissue regeneration. Despite its unique advantages, this novel therapeutic approach faces challenges owing to poor MSC survival rates and limited directed differentiation upon in vivo transplantation. In recent years, rapid developments in tissue engineering and biomaterials have led to the development of novel skin materials, including acellular dermal matrix (ADM), hydrogels, and silk proteins. Thus, these innovative materials are promising candidates for wound healing and regeneration. Biomaterials can be used not only as grafts to directly fill and repair tissue defects but also as vehicles to enhance the effectiveness of other therapies. Various studies have shown that combining MSCs with suitable carriers can increase their survival and promote the secretion of bioactive molecules, thereby promoting wound healing [51]. For local applications, combining cell-free substrates or scaffolds can enhance cell homing, differentiation, mobilization, and adhesion. Other researchers have investigated genetic modifications and pretreatment methods to enhance the therapeutic potential of transplanted MSCs.

### 5.1. Biological Scaffolds Combined With MSCs Promote Skin Damage Repair

For local applications, combining cell-free substrates or scaffolds can enhance cell homing, differentiation, mobilization, and adhesion. Additionally, genetic modifications and pretreatment have been explored by other researchers to further enhance the therapeutic potential of transplanted MSCs. However, there are challenges to realizing the full therapeutic potential of stem cells. To overcome these obstacles, additional strategies are necessary for optimizing MSC applications. Numerous experimental studies have demonstrated that transplanting MSCs into biomaterials enhances wound healing by localizing cells at the defect site and upregulating paracrine factors, thereby surpassing the efficacy of separate-cell applications. The subsequent Table 1 summarizes experimental investigations on the combined use of biomaterials and MSCs for burn wound treatment. Combining commonly used biomaterials, such as hydrogels, ADM, platelet-rich plasma (PRP), and nanofibers, with MSCs addresses the issue of inherent biological inactivity of the materials. By integrating MSCs, these biomaterials create a more favorable microenvironment for cell survival, promoting both proliferation and differentiation. For instance, Dong et al. [66] highlighted that chitosan hydrogels alone lack proangiogenic activity. However, when loaded with MSCs, it becomes proangiogenic and enhances tissue repair. Similarly, Mehrabani et al. [75] demonstrated that combining human Wharton's MSCs with ADM not only reduced cell apoptosis but also yielded positive therapeutic outcomes in mouse burn models. These findings underscore the potential of MSC-biomaterial combinations for effective burn wound treatment [75]. Wang et al. [81] explained the remarkable potential of combining ADSCs with collagen/PRP scaffolds. When transplanted into a full-thickness skin defect mouse model, this combination enhanced keratinocyte and fibroblast proliferation, facilitated vascular formation, and accelerated wound healing. These findings underscore the promising role of composite approaches in tissue regeneration [81]. Recently, several researchers explored the potential of combining modified biomaterials with MSCs for wound treatment. Modified biological scaffolds not only preserve the favorable biological properties of the original material but also introduce additional features that enhance cell growth and expedite wound healing. For instance, Alemzadeh, Oryan, and Mohammadi [65] investigated the combination of an ADM with human amnion (HA), along with loaded ADSCs. Their findings revealed that ADM-HA/ASCs significantly accelerated healing compared to that in wounds treated with ADM-HA or ADM alone. This innovative approach holds promise for advancements in wound repair [65]. Some studies have combined 3D bioprinting technology with biomaterials to create bioinks and load the corresponding cells for application in burn wounds, as the 3D bioprinting technology can optimize the cell compatibility of biomaterials and enhance their biological functions. An innovative approach by Roshangar et al. [73] which involved combining ADSCs with a 3D bioprinter-derived gel scaffold, yielded significantly superior results than those obtained using the 3D bioprinter-derived gel scaffold alone. Their findings underscore the potential of this combination for enhancing wound treatment [73].

### 5.2. Enhancing the Therapeutic Potential of MSCs Through Pretreatment

To enhance the therapeutic potential of transplanted MSCs, researchers have explored genetic modification and various pretreatment strategies. These approaches include drug administration, chemical exposure, trophic factor stimulation, cytokine stimulation, and hypoxia. The biochemical and biophysical properties of MSCs can be improved by certain pretreatments, thereby enhancing their repair functions. In vitro studies have demonstrated that the pretreatment of ADSCs with endothelial cell culture medium enhances their angiogenic capacity, proliferation, and differentiation toward the endothelium. This effect was also observed in a diabetic mouse model [82]. MSCs can also be pretreated with several chemicals to improve their ability to repair skin damage. For example, the treatment of ADSCs with lecithin-emulsified emu oil and butylated hydroxytoluene significantly increased their regenerative potential [83]. Attempts have also been made to activate UC-MSCs with bioglass materials to improve the healing capacity of mouse skin by stimulating paracrine effects between MSCs and receptor cells, and between fibroblasts and endothelial cells [84]. The application of curcumin as a pretreatment for MSCs, followed by transplantation onto wound surfaces, has demonstrated promising effects on skin wound healing. Specifically, curcumin enhanced the proliferation of BM-MSCs and modulated the composition and balance of fibronectin and collagen types I and III within the ECM of mice. Thus, this treatment approach leads to epidermal thickening and collagen deposition, closely resembling to those in normal skin [85]. Pretreatment with MSCs improves their differentiation ability, homing capacity, survival rate, and paracrine effects, ultimately accelerating wound healing. Srifa et al. [86] demonstrated that genetically modified MSCs secrete elevated levels of PDGF-BB and VEGF-A and that these factors contributed to enhanced wound healing following MSC implantation in a mouse model. In a rat model of deep second-degree burns, Aslam et al. [87] showed that pretreatment with isorhamnetin enhanced the survival and migration efficiency of UC-MSCs, ultimately promoting wound healing. Azam et al. [88] demonstrated that curcumin pretreatment of ADSCs promoted their proliferative, migratory, and paracrine potential, which may reduce macroinflammatory cell infiltration and promote collagen deposition and granuloma formation in acid-burned rats. Studies have indicated that modified MSCs exhibit a superior therapeutic efficacy than the unmodified MSCs. Among the various cell modification approaches, pretreatment appears to be the most clinically feasible and reproducible. Additionally, experimental evidence suggests that hypoxia enhances the wound-healing capacity of MSCs. In a mouse wound-healing model, human amniotic mesenchymal stem cells (hAMSCs) cultured under hypoxic conditions exhibited increased viability, proliferation, and VEGF expression relative to MSCs cultured under normoxic conditions. Furthermore, these hypoxia-conditioned MSCs enhanced the viability and migration of human dermal fibroblasts, upregulated ECM, and accelerated wound healing [89]. BM-MSCs appear to exhibit enhanced paracrine effects when exposed to hypoxic conditions. In vitro experiments have demonstrated increased secretion of bFGF, VEGF-A, and IL-6 by MSCs under hypoxic conditions [90]. Moreover, in vivo transplantation of BM-MSCs leads to accelerated wound contraction, cell proliferation, and neovascularization in mouse skin, all of which are attributed to the beneficial effects of hypoxia [91]. These results highlighted the potential of MSC-based therapies for wound healing, particularly when harnessed in hypoxic microenvironments. Genetic recombination of MSCs to use them as both seed cells and vectors to deliver target genes to the wound site is also a promising method of treating skin injuries. Several studies have targeted MSCs for genetic modification to enhance their ability to repair skin injuries by altering their activity. For instance, the overexpression of TGF*β*3 in BM-derived MSCs not only enhanced wound healing in rabbit ear tissue but also mitigated scar tissue formation, potentially preventing excessive scarring [92]. The researchers used recombinant lentiviral vectors to enhance IL-10 expression in human amniotic membrane-derived MSCs. They observed that these modified MSCs exhibited significantly greater efficacy than the unmodified MSCs across multiple wound-healing parameters in mice, including accelerated healing, enhanced angiogenesis, inflammation modulation, ECM remodeling regulation, and overall healing quality improvement [93]. Enhancing the reparative abilities of MSCs via genetic modification holds great promise for treating skin injuries. By fine-tuning their functions, we can improve wound healing outcomes and minimize scarring.

## 6. Clinical Trials of MSCs

Stem cell therapies have the potential for burn wound healing. These therapies can lead to reduced burn area, faster healing, and can prevent scar contraction. Researchers continue to explore innovative approaches to optimize stem cell treatments for burn injuries. In 2005, Rasulov et al. [94] administered allogeneic BM-MSCs from two healthy donors to a patient with 40% burn area. The study observed favorable wound kinetics, and after a few days of skin autografting, complete wound closure was achieved [94]. Mansilla et al. [95] investigated MSCs in the peripheral blood of patients with acute burns and healthy blood donors. They found that the percentage of MSCs correlated with burn size and severity. Moreover, higher percentages of MSCs were observed in younger patients. These findings highlight the potential role of MSCs in burn healing and warrant further exploration for therapeutic applications [95]. Lataillade et al. [96] documented the remarkable case of a patient with severe radiation-induced burns. Traditional surgical approaches, including excision, skin autografts, and flaps were ineffective. However, an innovative treatment strategy that combines surgery and local cellular therapy using autologous MSCs has been successful. The patient received multiple local MSCs along with skin autografts. Moreover, this combined treatment led to favorable clinical outcomes, with no recurrence of radiation-induced inflammatory waves during the 11-month follow-up period. The therapeutic potential of MSCs for severe radiation burns offers a promising strategy for wound repair and management [96]. In a 2010 case report, a patient with radiation burns received combination treatment. Autologous MSCs were injected into the affected area, along with autologous skin grafting. This approach led to favorable clinical progression, and there was no recurrence of radiation-induced inflammatory waves during the 8-month follow-up period. The use of MSCs in combination with surgical techniques holds promise for managing radiation burns [97]. In 2017, an Egyptian professor published a case-control study on the treatment of deep burns using BM-MSCs, UC-MSCs, and conventional therapy, which showed that BM-MSC and UC-MSCs could reduce early and late complications in patients and the length of hospital stay, effectively promoting wound healing [98]. In Jeschke et al.'s [99] study, BM-MSCs were administered to 70% of patients with burns in a patient-to-patient setting. This treatment promoted wound healing without complications and resulted in no scarring over the 6-year follow-up period. The use of BM-MSCs holds substantial promise for burn wound management [99]. Kitala et al. [100] successfully treated a 40-year-old woman with a deep burn (37%) using ADM-complexed hAMSCs. The wound healed completely within 12 days, and no scarring was observed [100]. Schulman et al. [101] divided 10 patients with deep second-degree burns into two groups—one group was administered 2.5 × 10³ BM-MSC/cm^2^ and the other was administered 5 × 10³ BM-MSC/cm^2^. All patients responded favorably to the treatment, the wounds were completely closed, no adverse effects were observed, and the effect was dose-dependent [101]. The absence of serious side effects in previous clinical studies suggests that stem cell therapy for patients with burns is a safe and effective approach. However, owing to variations in the type (UC, BM, and adipose-derived) and source of MSCs (autologous or allogeneic), a direct comparison of case study reports remains challenging. By December 2023, the largest clinical trial registry of the National Institutes of Health (http://clinicaltrials.gov) contained 23 registered clinical research projects focusing on stem cell treatment for burns. The results of these trials are listed in Table 2. In China, several hospitals have reported the use of stem cell treatments for burns. Moreover, the First People's Hospital of Zhengzhou City completed a “randomized controlled clinical study on human placental MSCs” in 2020. This study focused on treating wounds in the medium- and thick-skin donor areas of burn victims. The hospital officially launched this clinical study to provide valuable insights into stem cell therapy. At the time of compiling the paper, MSC products had been approved for marketing in the treatment of GVHD, knee osteoarthritis, Crohn's disease, and severe limb ischemia. Despite the paucity of clinical studies on the use of MSC in burn wounds, available data indicate promising outcomes. MSC-based therapies for wound repair are expected to be approved in the near future.

## 7. Conclusion

MSCs have been used to treat burn wounds and have shown better results than the conventional treatment methods. These cells promote neovascularization, reduce inflammation during wound healing, and lead to favorable outcomes, including minimal scar proliferation and improved sweat function. However, the main challenges are the low implantation and retention rates of MSCs. Researchers have proposed the use of bioscaffolds as an effective strategy for targeted stem cell delivery. Bioscaffolds provide structural support and create a biofunctional microenvironment that is conducive to cell adhesion, migration, and proliferation. This reduces cell damage due to external factors such as reactive oxygen species and improves the engraftment efficiency and survival time of MSCs. However, these biosynthetic materials merely represent the rudimentary replication of the cellular growth microenvironment. Consequently, further research is required to develop biomaterials that can meticulously regulate the growth, differentiation, survival, and ECM synthesis of MSCs, which are important for subsequent MSC applications.

## Figures and Tables

**Table 1:** Biological scaffolds combined with MSCs promote skin damage repair.

Study	Types of materials	Source of MSCs	Animal model	Types of skin damage	Effect
Liu et al. [52]	Collagen-GAG scaffolds	BM-MSCs	Pigs	Third-degree burn	Promoting vascularization optimizing epidermal formation and minimizing wound contraction
Mansilla et al. [53]	IADMs	BM-MSCs	Pig	Full-thickness burn	Successful wound healing led to complete closure and full skin regeneration, accompanied by minimal scarring
Hamrahi et al. [54]	Integra	ESC	Mice	Full-thickness burn	ESCs have demonstrated the ability to thrive both in vitro and in vivo on the Integra scaffold
Shokrgozar et al. [55]	Collagen chitosan	ADSCs	Rats	Full-thickness burn	The capacity of ASC in differentiation to keratinocytes and also wound healing in vivo
Souza et al. [56]	Nanostructured membrane	ADSCs	Rats	Second-degree burns	Enhance tissue repair
Steffens et al. [57]	Nanofiber scaffold	MSCs from mice kidney	Mice	Third-degree burn	Reducing validation response promotes wound repair
Yang et al. [58]	Fibrin glue	BM-MSCs	Rats	Full-thickness burn	Facilitate the regeneration of a fully functional skin structure and enhance the rate of healing in burned skin
Chung et al. [59]	P-fibrin	ASCs	Rats	Full-thickness burn	Promote neovascularization and accelerate wound healing
Ahmed et al. [60]	PRP	BM-MSCs	Rats	Full-thickness burn	Accelerate wound healing and promote MSCs proliferation
Alapure et al. [61]	ACgels	BM-MSCs	Mice	Third-degree burn wounds	Facilitated wound closure, reepithelialization, granulation tissue formation, and vascularization in burn wounds
Burmeister et al. [62]	FPEG hydrogels mitigate	ASCs	Pigs	Deep partial-thickness burn wounds	Minimize donor sites, accelerate healing, and improve outcomes
Oryan et al. [63]	DBM—*Aloe vera*	ASCs	Rats	Full-thickness burn	Promote wound surface and reduce scar formation
Qi et al. [64]	DADM	BM-MSCs	Mice	Full-thickness skin	Promoted wound healing in terms of angiogenesis, reepithelialization, and skin appendage regeneration
Alemzadeh, Oryan, and Mohammadi [65]	ADM-HA	ADSCs	Rats	Full-thickness burn	Diminished inflammation, enhanced angiogenesis, and improved granulation tissue formation
Dong et al. [66]	PEG-HA-RGD hydrogels	ADSCs	Mice	Deep second-degree burn	Augmented neovascularization, facilitated wound closure, and mitigated scar formation
Lu et al. [67]	mTG	ADSCs	Mice	Full-thickness burn	The cell spheroid has the possibility of cell–cell signaling to promote vascular generation
Nazempour et al. [68]	ADM	hWJSC	Rats	Third-degree burn	Reduce inflammation and improve wound healing
Barrera et al. [69]	Pullulan–collagen hydrogels	ASCs	Mice	Partial thickness contact burn	Accelerated healing, increased dermal appendage count, and improved scar quality with a more reticular collagen pattern
Costa de Oliveira Souza et al. [70]	Nanostructured bacterial cellulose-based membranes	ADSCs	Rats	Deep second-degree burn	Increased vascular proliferation and collagen deposition
Ng et al. [71]	Pristine gellan gum-collagen interpenetrating network hydrogels	ADSCs	Mice	Full-thickness burn	Enhance early wound closure, mitigating inflammation, and promoting complete skin regeneration
Paramasivam et al. [72]	PAUBM	BM-MSC	Rats	Full-thickness burn	Acceleration of angiogenesis and tissue regeneration
Roshangar et al. [73]	3D bioprinter-derived-gel scaffold	ADSCs	Rats	Full-thickness burn	Epithelization was faster and formed a multilayered epidermis
Sharifi et al. [22]	*A. vera* gel and chitosan-based gel	BM-MSCs	Rats	Second-degree burn	Highest angiogenesis and granulation tissue formation
Wu et al. [74]	GS alginate hydrogels	ADSCs	Mice	Full-thickness burn	Promote severe burn wound healing through increased neovascularization via the VEGF signaling pathway

Mehrabani et al. [75]	ADM	hWJSC	Rats	Third-degree burn	SPION labeling, combined with MRI, enables deeper insights into the behavior and destiny of stem cells following transplantation in a burn injury model
Naasani et al. [76]	DhAM	ADSCs	Mice	Second-degree burns	Reduced inflammation
Tammam et al. [77]	PRP	BM-MSCs	Rats	Full-thickness burn	Improvement in burn healing by increasing the contraction rate, burn area, and period of epithelization
Yu et al. [78]	Zwitterionic polysaccharide-based hydrogel	ADSCs	Mice	Deep second-degree burns	Enhancing M2 polarization of macrophages promotes collagen deposition and stimulates angiogenesis
Zhou et al. [79]	mAM	UC-MSC	Mice	Deep second-degree burns	Accelerated wound healing and prolonged MSC survival, resulting in enhanced outcomes
Nikzad et al. [80]	B-AOSIS	WJ-MSCs	Rats	Third-degree burns	Boron (B) enriched-acellular sheep small intestine submucosa

*Note:* ACgels, unsaturated arginine-based poly(ester amide) (UArg-PEA) and chitosan derivative; B-AOSIS, boron enriched-acellular sheep small intestine submucosa; DSC; B-AOSIS, boron enriched-acellular sheep small intestine submucosa; mTG, gelatin/Mtg hydrogel; P-fibrin, PEGylated fibrin.

Abbreviations: DADM, denatured acellular dermal matrix; DBM, demineralized bone matrix; DhAM, decellularized human amniotic membrane; ESC, embryonic stem cells; FPEG, polyethylene glycol; GAG, glycosaminoglycan; HA, hylauronic acid; IADMs, intelligent acellular dermal matrices; PAUBM, porcine acellular urinary bladder matrix; PRP, platelet rich plasma; WJ-MSCs, Wharton's jelly-derived mesenchymal stem cells.

**Table 2:** Clinical application of MSCs.

Study	Type	Patients	Methods	Outcome
Rasulov et al. [94]	Case report	A female patient presented with extensive skin burns (I-II-IIIAB degree skin burns, with a total area of 40% and an area of IIIB degree reaching 30%)	Making use of the transplantation of allogenic fibroblast-like BM-MSCs onto the surface of deep thermal burns	Quicker healing of donor zones and accelerated recovery of the patient

Mansilla et al. [95]	Clinical study	The blood acquired from burn patients presented a greater MSC percentage	Flow analysis of the blood	The blood sampled from burn patients displayed a higher MSC percentage
Lataillade et al. [96]	Case report	On December 15, 2005, a 27-year-old Chilean man was overexposed to a gammagraphy radioactive source (192Ir, 3.3 TBq)	Merged numerical dosimetry-guided surgery with cellular therapy involving mesenchymal stem cells	During the 11-month patient's follow-up, no recurrence of radiation inflammatory waves was noted
Bey et al. [97]	Case report	A 32-year-old male patient was excessively exposed to a gammagraphy radioactive source (192Ir, 2.2 TBq) for 17 h.	A sum of five local MSC administrations were conducted in combination with skin autograft	The clinical progress was favorable and no recurrence of radiation inflammatory waves happened during the 8-month follow-up of the patient
Abo-Elkheir et al. [98]	Prospective study	Thermal full-thickness percentage ranging from 10% to 25% total body surface area (TBSA)	BM-MSCs or UC-MSCs or the conventional early excision and graft (EE&G) for thermal full-thickness burned patients	BM-MSCs or UC-MSCs or conventional EE&G among thermal full-thickness burned patients
Jeschke et al. [99]	Case report	A male having ≥70% TBSA burns, mainly full thickness, and sustained smoke inhalation injury	Given treatment with allogeneic MSCs	Wound healing was speeded up with no adverse treatment complications. Wound sites displayed no evidence of keloids or hypertrophic formation during a 6-year follow-up period
Schulman et al. [101]	Clinical trial	Ten individuals who are 18 years old or older and have deep second-degree burn wounds	The first five patients were given 2.5 × 10³ BM-MSC/cm^2^ to their wounds, and a second group of five patients was treated with a higher concentration of 5 × 10³ allogeneic BM-MSC/cm^2^	All patients responded favorably to the treatment, attaining 100% closure of wounds and presenting minimal clinical evidence of fibrosis

Abbreviations: BM, bone marrow; MSCs, mesenchymal stem cells.

## Data Availability

Data used to support the findings of this study are available from the corresponding author upon request.
